# Pioglitazone Mediates Cardiac Progenitor Formation through Increasing ROS Levels

**DOI:** 10.1155/2022/1480345

**Published:** 2022-09-10

**Authors:** Maryam Baharlooie, Maryam Peymani, Mohammad Hossein Nasr-Esfahani, Kamran Ghaedi

**Affiliations:** ^1^Department of Cell and Molecular Biology and Microbiology, Faculty of Biological Science and Technology, University of Isfahan, Isfahan, Iran; ^2^Department of Biology, Faculty of Basic Sciences, Shahrekord Branch, Islamic Azad University, Shahrekord, Iran; ^3^Department of Animal Biotechnology, Cell Science Research Center, Royan Institute for Biotechnology, ACECR, Isfahan, Iran

## Abstract

In order to achieve a sufficient population of cardiac-committed progenitor cells, it is crucial to know the mechanisms of cardiac progenitor formation. Previous studies suggested ROS effect on cardiac commitment events to play a key role in the cell signaling and activate cardiac differentiation of pluripotent stem cells. We previously reported that PPAR*γ* activity is essential for cardiac progenitor cell commitment. Although several studies have conducted the involvement of PPAR*γ*-related signaling pathways in cardiac differentiation, so far, the regulatory mechanisms of these signaling pathways have not been discussed and cleared. In this study, we focus on the role of PPAR*γ* agonist in ROS generation and its further effects on the differentiation of cardiac cells from mESCs. The results of this study show that the presence of ROS is necessary for heart differentiation in the precursor stage of cardiac cells, and the coenzyme Q10 antioxidant precludes proper cardiac differentiation. In addition, this antioxidant prevents the action of pioglitazone in increasing oxygen radicals as well as beating cardiomyocyte differentiation properties. In this case, it can be concluded that PPAR*γ* is required to modulate ROS levels during cardiac differentiation.

## 1. Introduction

Given the increased rate of cardiovascular diseases worldwide, along with the consequential burden of heart failure cares, there is a growing request for cardiac regeneration with functional cardiomyocytes [1], as well as a robust generation of cardiac cell models for drug discovery studies [2]. Due to the proliferative and integrative capacity of progenitor cells, as well as their commitment to the cardiac cell fate and safety in the host tissue, these cells are more suitable than stem cells for cell therapy approaches [3]. In order to achieve a sufficient population of cardiac-committed progenitor cells, it is essential to know the mechanisms of cardiac progenitor formation. Previous studies suggested ROS effect on cardiac commitment events to play a key role in the cell signaling and activate cardiac differentiation of pluripotent stem cells [4].

Peroxisome proliferator-activated receptors (PPARs) as a superfamily members of nuclear receptors are ligand-activated transcription factors. PPAR*γ* is activated by natural fatty acids (primarily unsaturated fatty acids) or synthetic agonists, including pioglitazone (Pio). PPARs play critical roles in the pathophysiology of cardiovascular diseases, such as energy balance, cell proliferation, apoptosis, and adipocyte differentiation [5]. For instance, by decreasing PPAR*α* activity, the expression of cardiac sarcomeric proteins and specific genes is reduced, and finally, cardiac differentiation is inhibited [6]. PPAR*γ* is involved in the regulation of differentiation and homeostasis, including adipocyte differentiation, insulin sensitivity, anti-inflammatory activities, and cell proliferation [5].

Although several studies have conducted the involvement of PPAR*γ*-related signaling pathways in cardiac differentiation, so far, the regulatory mechanisms of these signaling pathways have not been discussed and cleared. We previously reported an essential role of PPAR*γ* activity in the cardiac progenitor cell commitment [7].

Also, we have investigated its protective role in doxorubicin-induced cardiomyopathy in a chemotherapy model of differentiated cardiac cells [8].

In this study, in order to identify the mechanism of PPAR*γ* function, we focus on the role of PPAR*γ* agonist in ROS generation and its further effects on the differentiation of beating cardiomyocytes from mESCs.

## 2. Methods

### 2.1. Cell Culture and Differentiation

Mouse embryonic stem cell line RB20 supplied from Royan Institute was cultured and expanded in 0.1% gelatin-coated adherent plates at 37°C under 5% CO_2_. Cells were maintained in an undifferentiated state in Aston Smith medium as previously reported [9].

Cardiac differentiation was performed using hanging drop method with 8∗10^2^ cells per each 20 *μ*L drop. After the formation of embryoid bodies (EBs), they were transmitted to nonadhesive cell culture dishes in a suspension culture system to obtain cardiac progenitor cells (Greiner, Germany; 628102). Ascorbic acid 10 *μ*M was used to improve spontaneous differentiation to cardiac progenitors. On day 7, cardiac progenitor EBs were plated on 0.1% gelatin-coated adherent dishes and, on days 14 and 7, the beating cardiomyocytes were collected in TRIzol reagent (Ambion).

### 2.2. Treatments

Pioglitazone (5, 10, and 20 *μ*M) (Cayman), GW9662 (10, 20 *μ*M) (Cayman), a-lipoic acid (50, 200, and400 *μ*M), and coenzyme Q10 (CoQ10) (0.5, 1, and 5 *μ*M) were used for assessment of ROS generation. We optimized the concentration of each component individually in the production of ROS, by measuring fluorescence intensity using fluorescent microscopy, as well as flow cytometry.

### 2.3. ROS Generation Measurement

**Figure 1:**
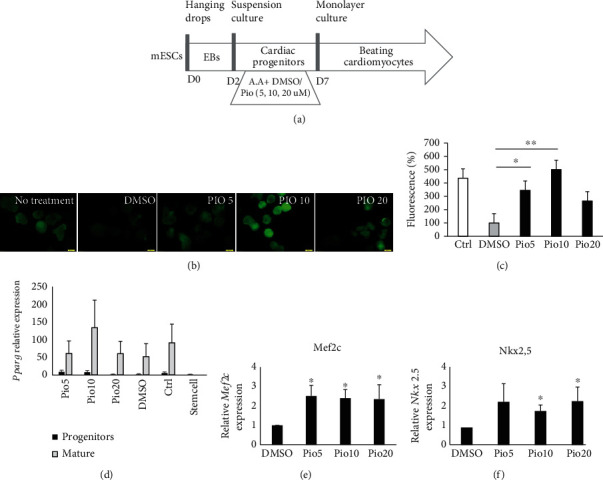
Percentage of fluorescence and gene expression in treatment with different concentrations of pioglitazone. (a) Illustrated protocol of mESCs to beating body differentiation. (b) Fluorescent imaging of beating bodies after differentiation of EBs treated in different concentrations of Pio, on day 7. (c) Percentages of fluorescence intensity in different concentrations of Pio, on day 7. (d) *Pparg* relative expression in stem cells, progenitors, and mature EBs in different concentrations of Pio, on day 7. (e) *Mef2c* relative expression in progenitors in different concentrations of Pio, on day 7. (f) *Nkx2,5* relative expression in progenitors in different concentrations of Pio, on day 7. Represented value bars are the mean of triplicate independent experiments ± SEM (*P* value < 0.05). Scale bar is 200 mm.

We adjusted the selected doses of each antioxidant and Pio treatment according to the test conditions and culture medium (such as vitamin C levels set for cardiac differentiation, which is itself an antioxidant). To set the doses as well as measuring ROS generation in the presence of each treated reagent, fluorescent DCF staining was used in the 6^th^ day of cardiac progenitor differentiation in suspension culture (Figure 1(a)). Fluorescence microscopy was used to capture pictures, and ImageJ software was used for the quantification of fluorescence density. The fluorescence density was normalized and calculated using the corrected total cell fluorescence (CTCF) formula as CTCF = integrated density–(area of selected cell × mean fluorescence of background readings).

### 2.4. Bioinformatics Studies

In the first step, we obtained a GEO dataset (GSE83428) regarding cardiac commitment of mESCs and analyzed it via Geo2R tool [10]. Then, we sorted significant (adjusted *P* value ≤ 0.01) differentially expressed genes (DEGs) with log fold change ≥ 2, by their upregulation or downregulation status. Also, we performed this process for the second dataset (GSE103560) regarding final cardiac differentiation of cardiac-committed progenitor cells. Then, we mapped the results of the first step to the second dataset and plotted a Venn diagram [11]. We selected a subset of genes based on our hypothesis on the potential biphasic involvement of signaling pathways such as Wnt signaling in cardiac differentiation steps. We then enriched this subset with their 20 top first-shell interacting proteins to the gene ontology (GO) biological process (BP) repository data. We then analyzed and visualized this network in Cytoscape *v*3.6 software [12].

### 2.5. Real-Time Quantitative PCR (RT-qPCR) Analysis

Total RNA extraction was carried out using TRIzol protocol [13], and cDNA synthesis was done using Takara cDNA synthesis kit. RT-qPCR was performed by an ABI instrument (Applied Biosystems, Foster, CA, USA) applying SYBR Green kit (TaKaRa). Primer pairs specific for candidate genes were designed using Beacon designer (version 7.2, USA) and were confirmed in oligo7 subsequently by blast in the NCBI database. Primer sequences are provided in Table 1. Relative expression levels of genes were assessed with the 2^–*ΔΔ*Ct^ method using glyceraldehyde 3-phosphate dehydrogenase (GAPDH) as the internal control.

### 2.6. Statistical Analysis

Statistical differences between groups were identified by Tukey's post hoc test with one-way analysis of variance and independent Student's *t*-test using IBM SPSS v23.0 and GraphPad Prism 6. All experiments were replicated at least in three independent experiments. Values are represented as mean ± SEM (standard error of the mean).

## 3. Results

### 3.1. Pioglitazone (Pio) Promotes ROS Generation in Cardiac Differentiation

As illustrated in the schematic experimental protocol, EBs were treated with different concentrations of pioglitazone for 5 days (Figure 1(a)). Analysis of fluorescence intensity using CTCF formula showed a significant increase in ROS intensity levels in the cardiac progenitor EBs under treatment with Pio 10 *μ*M (*P* value < 0.01) and Pio 5 *μ*M (*P* value < 0.05) compared to DMSO. Pio is a solute in DMSO reagent, and we must use DMSO group for control. So, both of the Pio5 and 10 *μ*M groups showed increase in ROS and also PPAR*γ* levels compared to DMSO. However, we chose Pio10 for further examinations, according to its best ROS production function (Figures 1(b) and 1(c)).

Expression levels of *PPARγ* were elevated in progenitor EBs compared to the stem cells, as well as in the beating cardiomyocytes in comparison to the progenitor EBs. This elevation was observed in all pioglitazone concentrations, with the most significant one in Pio 10 *μ*M (Figure 1(d)). Also, expression levels of cardiac progenitor markers, *Mef2c* and *Nkx2,5*, were upregulated in the presence of Pio 10 *μ*M. So, we continued the next steps of the experiment with Pio in the concentration of 10 *μ*M (Figures 1(e) and 1(f)).

### 3.2. Bioinformatics Study and Retrospective Literature Validation Support Evidences of Biphasic Regulation of ROS-Associated Gene Ontologies in Cardiac Differentiation Stages

**Figure 2:**
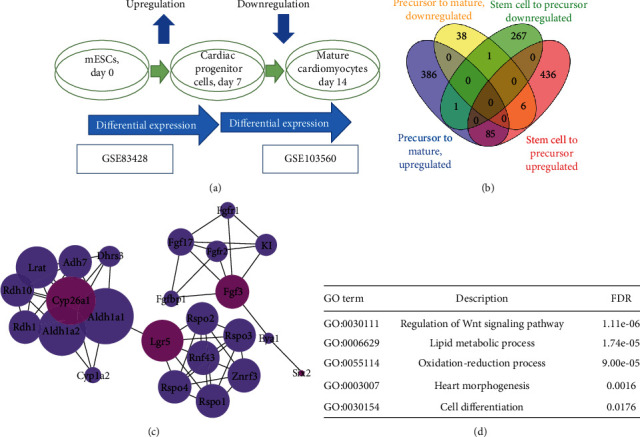
In the bioinformatics section of this study, two GSE83428 and GSE103560 data were selected, respectively, related to the differentiation of mouse embryonic stem cells into cardiac precursors and the distinction of precursor cells into adult heart cells. Genes with significant expression change (*P* < 0.005) and log fold change > 2 were screened and selected for the next step. (a) Selection of bioinformatics study data and illustrated protocol of mESCs to beating body differentiation in the microarray studies with differentiation protocols similar to our experimental process. (b) The classification of genes with significant expression change is the result of two data. (c) Expanded gene network resulting from biphasic expression analysis data. (d) The ontology of the enriched gene is expanded in the gene list.

We obtained 820 DEGs from analyses of GSE83428 and 2887 DEGs from analyses of GSE103560 datasets in which we selected 796 and 517, respectively, according to logFC ≥ 2 and adj.*P* value ≤ 0.01 criteria. The Venn diagram illustrates that 6 genes were altered in a biphasic pattern, consistent with Wnt signaling genes (Figures 2(a) and 2(b)). A network expansion followed by network analysis in Cytoscape network analyzer application revealed four of these genes (*Lgr5*, *Cyp26a1*, *FGF3*, and *Six2*) to participate in the network and three of them to be hub genes (Figures 2(b) and 2(c)). Gene ontology enrichment analysis of the resulted network showed that GO0030111, regulation of the Wnt signaling pathway; GO0006629, lipid metabolic process; GO0055114, oxidation-reduction process; GO0003007, heart morphogenesis; and GO0030154, cell differentiation were among top enriched gene ontologies (Figure 2(d)).

After that, by retrospective literature validation, we found that previously, LGR5 which is a well-known stem-cell-growth marker [14] was reported to be increased in the early stage of cardiomyocyte differentiation expression. Also, LGR5 is necessary for cardiomyocyte differentiation, and its knockdown leads to the fate of endothelial cells. Furthermore, knockdown of LGR5 downregulated the genes related to the Wnt signaling pathway [15].

Calderon et al. found that *Cyp26a1* is toughly increased on day 3 of early mesoderm differentiation and quickly decreased after that and lasts in low levels till the end of cardiac differentiation subsequently [16]. Localized expression of Cyp26 enzymes which metabolize retinoic acid levels in embryos warrants a proper balance between myocardial and endothelial lineages [17].

Furthermore, FGF3 mutations might contribute to congenital heart defects in human. Interestingly, Urness et al. previously demonstrated that the dosage of *Fgf3* is a sensitive factor in cardiac growth, and *Fgf3* normalizes expression of some key transcription factors of cardiac mesoderm [18].

Moreover, it is revealed that mammalian heart is developed consecutively by separate cells of cardiac progenitor, recognized as the first heart field and second heart field subpopulations. Zhou et al. demonstrated that Six2 is dynamically expressed in a subpopulation of the second heart field, and ablation of Six2+ progenitors at specific stages leads to congenital heart diseases [19].

### 3.3. Antioxidants Suppress Cardiac Progenitor Formation

**Figure 3:**
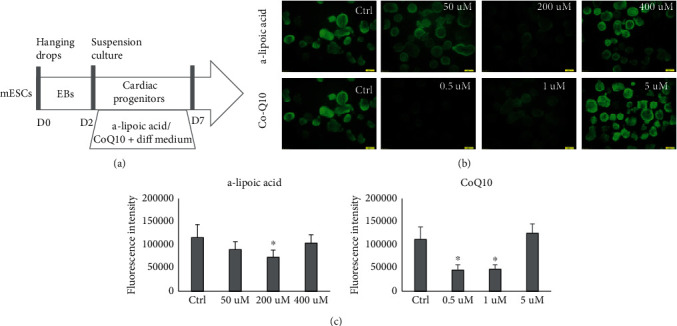
Percentage of fluorescence and gene expression in treatment with different concentrations of two types of antioxidants. (a) Illustrated protocol of mESCs to progenitors treated in different concentrations of a-lipoic acid and CoQ10, on the day7. (b) Fluorescent imaging and intensities (c) of beating bodies after differentiation of EBs treated in different concentrations of a-lipoic acid and CoQ10, on day 7. Scale bar is 200 mm.

In this step, EBs were treated with either a-lipoic acid or coenzyme Q10 during days 2 to 7 of differentiation (Figure 3(a)). Evaluation of ROS level via fluorescent microscopy showed a decrease in ROS intensity in the presence of either CoQ10 or a-lipoic acid (Figures 3(b) and 3(c)).

### 3.4. Antioxidants Inhibit Pioglitazone Effects on Cardiac Differentiation

**Figure 4:**
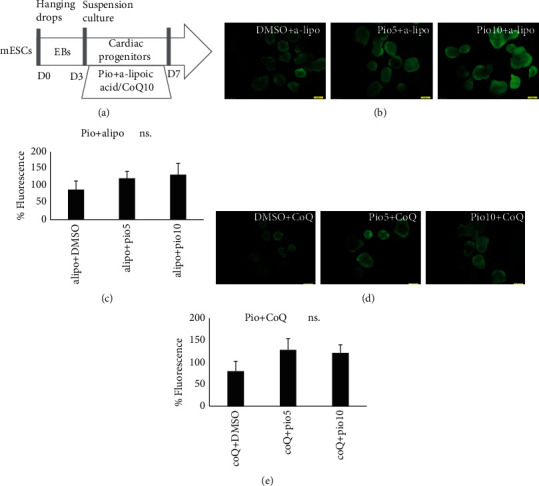
Percentage of fluorescence and gene expression in treatment with pioglitazone in combination with two types of antioxidants. (a) Illustrated protocol of mESCs to progenitors treated in different concentrations of a-lipoic acid and CoQ10, on the day7. (b) Fluorescent imaging and intensities (c) of beating bodies after differentiation of EBs treated in different concentrations of a-lipoic acid along with Pio, on day 7. (d) Fluorescent imaging and intensities (e) of beating bodies after differentiation of EBs treated in different concentrations of CoQ10 along with Pio, on day 7. Scale bar is 200 mm.

Changes in ROS levels in the presence of the combination of pioglitazone treatment with a-lipo nor CoQ10 (Figure 4(a)) were not significant compared to those of DMSO+a-lipo (Figures 4(b) and 4(c)) or DMSO+CoQ10 (Figures 4(d) and 4(e)), respectively.

**Figure 5:**
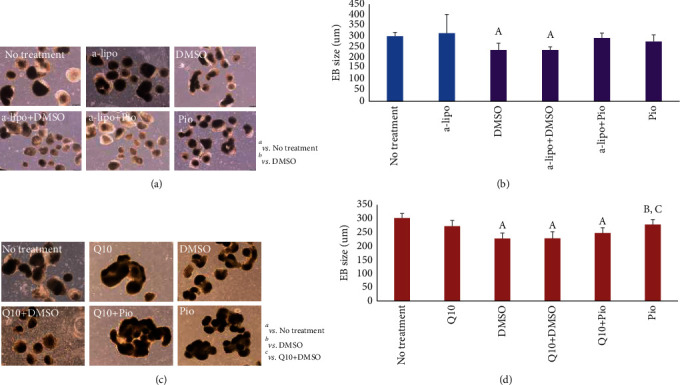
Measurement of embryoid body-derived precursors of the heart in the treatment with pioglitazone in combination with two types of antioxidants. Morphological illustration (a) and EB size (*μ*m) (b) of generated mESC-derived progenitors treated with antioxidant a-lipoic acid and morphological illustration (c) and EB size (*μ*m) (d) of generated mESC-derived progenitors treated with CoQ10, both in the presence and absence of Pio, on day 7. Alphabets indicate significant difference between samples at *P* value < 0.05. A represents significance vs. no treatment and B vs. DMSO. Scale bar is 200 *μ*m.

Progenitor EB sizes were decreased in the presence of DMSO and increased significantly under Pio treatment in a-lipo or CoQ10 set. Progenitor EB sizes in the presence of Pio were significantly increased compared to those of DMSO or DMSO+CoQ10. However, under Pio+CoQ10 treatment, progenitor EBs were similar in size to Pio treatment alone (Figure 5). This result, in addition to the nonsignificant elevation of ROS in Pio+CoQ10 (Figure 4), suggested that ROS could not be generated under Pio treatment in the presence of CoQ10 antioxidant.

**Figure 6:**
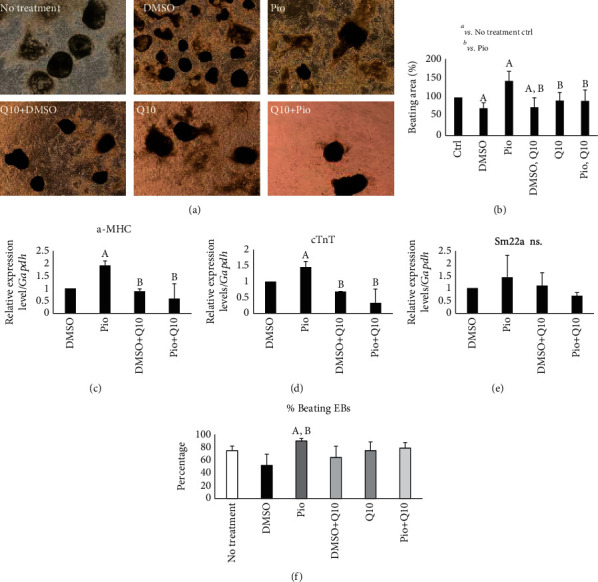
Beating area, gene expression, and percentage of embryoid body-derived precursors converted to beating heart objects in mature heart cells treated with pioglitazone in combination with two types of antioxidants. Morphological illustration (a) and beating area percentages (b) of generated mESC-derived beating EBs treated with antioxidant CoQ10 along with Pio, on the day 14. Scale bar is 200 mm. Relative expression levels of a-MHC (c), cTnT (d), and Sm22a (e) in beating EBs treated with CoQ10 along with Pio, on day 14. Represented value bars are the mean of triplicate independent experiments ± SEM (*P* value < 0.05). Alphabets indicate significant difference between samples at *P* value < 0.05. A represents significance vs. no treatment and B vs. Pio. (f) Percentages of beating EBs treated with antioxidant CoQ10 along with Pio, on the day 14.

Also, Pio enhanced the beating properties of EBs, such as percentage of beating EBs and beating area, while CoQ10 antioxidant decreased these properties (Figures 6(a) and 6(b)). Mature cardiac markers, *a-Mhc* and *cTnT*, were upregulated under Pio treatment and downregulated in the presence of Pio+CoQ10 as well as DMSO+CoQ10 (Figures 6(c) and 6(d)) while the expression level of *Sm22a* as a smooth muscle marker did not change (Figure 6(e)).

### 3.5. PPAR*γ* Antagonist Inhibits Pioglitazone Effects on Cardiac Differentiation

We used GW9662 as a specific PPAR*γ* antagonist. To investigate the role of PPAR*γ* on cardiac differentiation, EBs were treated simultaneously with PPAR*γ* agonist and antagonist as depicted in supplemental Figure 1A (concentration setup data for antagonist were not shown). The results showed that beating EBs and EB sizes were altered by GW9662 (supplemental Figure 1B, C). Also, real-time PCR data indicated that the expression levels of a-MHC, cTnT, SM22a, and Pparg were downregulated by PPAR*γ* antagonist. Furthermore, Pio could not reverse this inhibition (supplemental Figure 1C-G).

## 4. Discussion

Use of pluripotent stem cell-derived cardiomyocytes in vitro is a potentially promising approach for regenerative medicine and drug screening models. Still, the inability to sufficiently produce cardiac cells with high quality has been a major restriction to apply this potential. Therefore, understanding the molecular switches that control cardiac commitment is essential to better understand the development of the heart and design better approaches to the treatment of heart diseases.

Although several studies have been performed to investigate the role of PPAR*γ* during development, limited information is available on the mechanism of its effect on the cardiac differentiation. In the present study, we suggest the role of this receptor in ROS generation process involved in cardiac differentiation of mouse embryonic stem cells.

The results of this study show that the presence of ROS is necessary for cardiac differentiation in the progenitor stage of cardiac cells, and the coenzyme Q10 antioxidant can prevent proper cardiac differentiation. In addition, this antioxidant prevents the effect of pioglitazone in increasing ROS as well as cardiac differentiation properties. In this case, it can be concluded that PPAR*γ* activation modulates ROS levels during cardiac differentiation. This is consistent with the bioinformatics findings of this study from analyzing microarray studies. In these findings, biphasic pathways in the process of cardiac differentiation include Wnt pathway regulation, oxidation-reduction, and gene ontologies related to cell differentiation and cardiac morphogenesis.

Excessive amounts of ROS could lead to oxidative stress and pathogenesis. However, balanced amounts of ROS play a key role in cell signaling and activate cardiac differentiation of pluripotent stem cells [4].

ROS is tempered via the Hippo signaling pathway with the involvement of oxidant/antioxidant processes, which affects cardiac cell proliferation [4]. Moreover, in the bioinformatics results, we observed that the mmu04390: Hippo signaling pathway is the most enriched pathway in the progenitor to mature differentiation stage, which has a decline in gene expression pattern in the GEO data. Also, mmu04550: signaling pathways regulating pluripotency of stem cells and mmu04310: Wnt signaling pathway were downregulated in the GEO data, supporting the idea of ROS-associated Wnt signaling decline while getting away from the pluripotency state in the progenitor formation. Also, many studies have shown the role of the Wnt pathway in cardiac development and differentiation from stem cells [20]. It is believed that Wnt/*β*-catenin signaling system plays crucial roles in developing embryos and also in adult stem cell self-renewal and maintenance [21].

Herrero et al. have reported that ROS levels are critical regulators of adult cardiac progenitor cell differentiation and are directly associated to cardiac-related gene expression in vivo [22].

Supporting our idea, it was demonstrated by Crespo et al. that catalase, N-acetyl cysteine, and mitoubiquinone antioxidants interfere with proper cardiac differentiation of embryonic stem cells. Also, ascorbic acid as a prooxidant agent compensates the shortage of ROS levels in low glucose media [23].

In contrast, Choe et al. showed a beneficial effect of antioxidants such as Trolox, an analog of vitamin E, in cardiac differentiation. This effect is through inhibition of the Wnt pathway. However, it has shown a time- and dose-dependent manner, and it works after the mesodermal differentiation stage, which interestingly supports the Wnt downregulation in the second stage of cardiac differentiation [24].

PPARs form a heterodimer with the retinoid X receptor (RXR) and regulate transcription of target genes through binding to their specific responsive elements (PPRE). PPARs control the expression of various gene networks, including well-known pathways such as adipogenesis, lipid metabolism, inflammation, and metabolic homeostasis maintenance [25]. In the bioinformatics part of this study, we identified two genes, *FGF3* and *six2*, harboring PPRE in the cell differentiation pathway enriched in the gene network related to cardiac progenitor differentiation.

We identified that Pio elevates ROS levels during cardiac progenitor formation. Results showed that the levels of ROS are decreased following a-lipo and CoQ10 antioxidant treatment. However, there is no significant change in the level of ROS in the presence of Pio combined with CoQ10 treatment (Figure 4). It is suggested that Pio could not generate ROS in the presence of CoQ10 antioxidant. In addition, it was observed that cardiac progenitor and mature markers are affected by Pio and CoQ10 antioxidant in the same trend as ROS levels. We previously reported that Pio is necessary for appropriate cardiac differentiation. In the present study, we showed that cardiac differentiation moderates in the presence of the combination of antioxidants and Pio. Pio treatment without ROS generation could not significantly affect the cardiac differentiation. Altogether, these results suggest that the role of Pio during cardiac differentiation depends on its ROS generation function and capability.

## 5. Conclusion

We identified that Pio elevates ROS levels in cardiac progenitor formation. Cardiac progenitor and mature markers are affected by Pio and CoQ10 antioxidant in the same trend as ROS levels. We previously reported that Pio is necessary for appropriate cardiac differentiation. In the present study, we showed that cardiac differentiation diminishes when antioxidants are applied in the same manner as when combining antioxidant with Pio. Altogether, these results suggest that the role of Pio in cardiac differentiation could be dependent on its ROS generation function.

Bioinformatics results also showed that signaling pathways regulating pluripotency of stem cells and Wnt signaling pathway were downregulated, supporting the idea of ROS-associated Wnt signaling modulation while getting away from the pluripotency state in the cardiac differentiation.

## Figures and Tables

**Table 1:** Primer sequences used for gene expression analysis.

Genes	Primer sequence (5′-3′)	Annealing temp (°C)	Accession no.
Gapdh	F: TGCCGCCTGGAGAAACC R: TGAAGTCGCAGGAGACAACC	58	NM_008084.2
PPAR*γ*	F: TGAGACCAACAGCCTGAC R: GTTCACCGCTTCTTTCAAATC	60	NM_001127330.1
Nkx2.5	F: TTAGGAGAAGGGCGATGAC R: AGGGTGGGTGTGAAATCTG	57	NM_008700.2
Mef2c	F: CGAGTGTAAGTGTCTAATG R: CCTATTGTCAGAATTGCTAT	54	NM_001170537.1
*α*-MHC	F: CAGAAGCCTCGCAATGTC R: CGGTATCAGCAGAAGCATAG	58	NM_001164171.1
cTnT	F: ACAGAGGAGGCCAACGTAGAA R: CTCTCTCCATCGGGGATTCTT	60	NM_001130181.2

## Data Availability

The datasets used and/or analyzed during the current study are available from the corresponding authors on reasonable request.

## Abbreviations

ANOVA: Two-way analysis of variance
GAPDH: Glyceraldehyde 3-phosphate dehydrogenase
GO: Gene ontology
PPAR*γ*: Peroxisome proliferator-activated receptor *γ*
RT-qPCR: Reverse transcription quantitative polymerase chain reaction.

## Supplementary Materials

Supplementary Materials Supplemental Figure1: EB size, gene expression, and percentage of beating embryoid bodies in mature heart cells treated with pioglitazone in combination with GW9662 as a specific PPAR*γ* antagonist. Illustrated protocol of mESCs to cardiomyocytes treated with Pio and GW9662 (A). Percentage of beating EBs (B), and EB size (um) (C) of generated mESC-derived embryoid bodies treated with Pio and GW9662. *a*-*MHC* relative expression in beating EBs treated with Pio and GW9662, on day 14 (D); *cTnT* relative expression in beating EBs treated with Pio and GW9662, on day 14 (E); *SM22a* relative expression in beating EBs treated with Pio and GW9662, on day 14 (F); *Pparg* relative expression in beating EBs treated with Pio and GW9662, on day 14 (G). Represented value bars are the mean of triplicate independent experiments ± SEM (*p* value < 0.05).
