# Supplementary material for: Pioglitazone Mediates Cardiac Progenitor Formation through Increasing ROS Levels
Source: Biomed Res Int. 2022 Sep 10;2022:1480345. doi: 10.1155/2022/1480345 (PMC9482506; doi:10.1155/2022/1480345)
Supplement: Supplementary Materials — Supplemental Figure1: EB size, gene expression, and percentage of beating embryoid bodies in mature heart cells treated with pioglitazone in combination with GW9662 as a specific PPARγ antagonist. Illustrated protocol of mESCs to cardiomyocytes treated with Pio and GW9662 (A). Percentage of beating EBs (B), and EB size (um) (C) of generated mESC-derived embryoid bodies treated with Pio and GW9662. a-MHC relative expression in beating EBs treated with Pio and GW9662, on day 14 (D); cTnT relative expression in beating EBs treated with Pio and GW9662, on day 14 (E); SM22a relative expression in beating EBs treated with Pio and GW9662, on day 14 (F); Pparg relative expression in beating EBs treated with Pio and GW9662, on day 14 (G). Represented value bars are the mean of triplicate independent experiments ± SEM (p value < 0.05). [file 1480345.f1.pptx]

## Slide 1
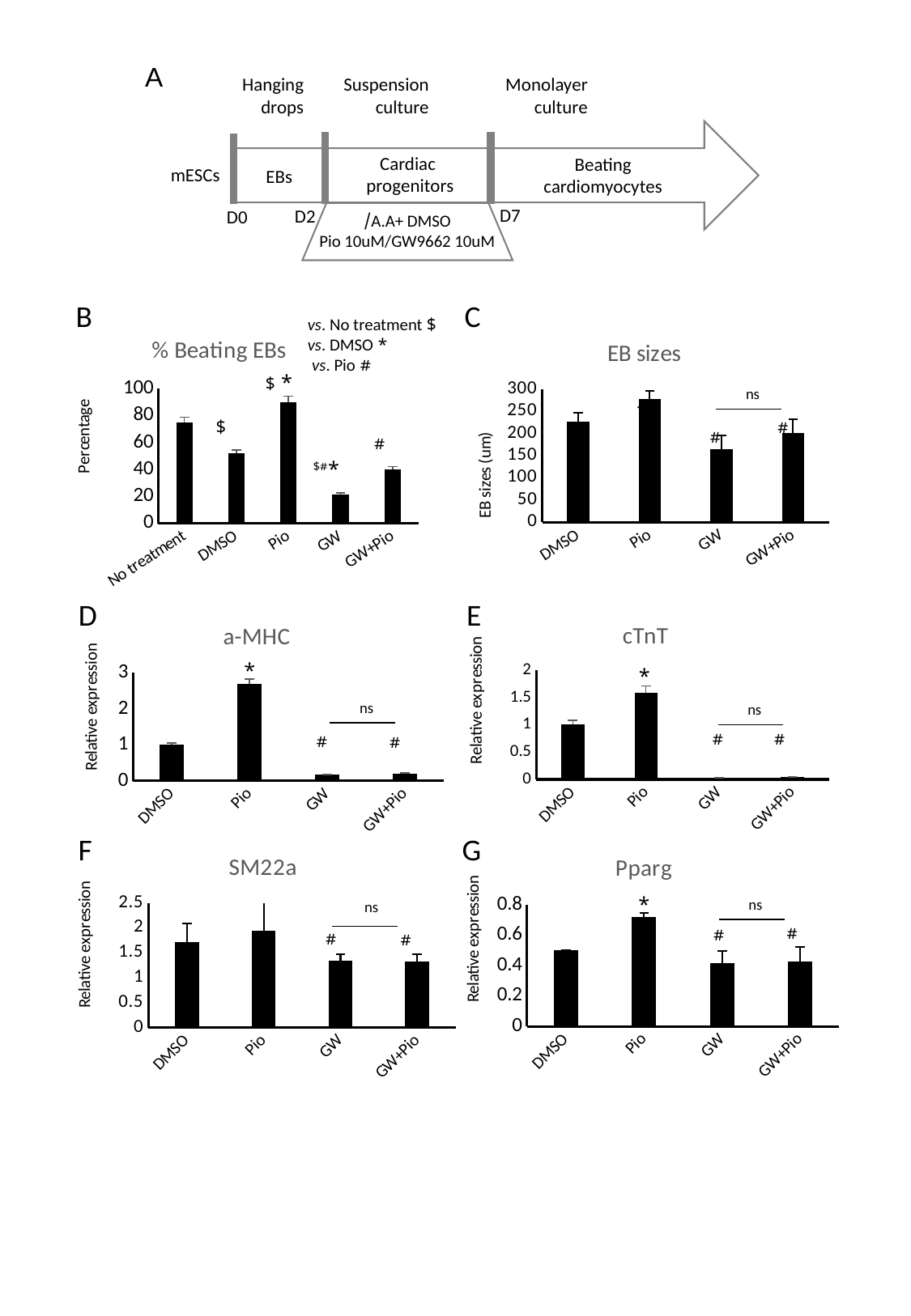

A
Hanging drops
Suspension culture
Monolayer culture
Cardiac progenitors
Beating cardiomyocytes
mESCs
EBs
D7
D2
D0
A.A+ DMSO/
Pio 10uM/GW9662 10uM
B
C
$ vs. No treatment
* vs. DMSO
# vs. Pio
### Chart: % Beating EBs
| Category | |
|---|---|
| No treatment | 75.0 |
| DMSO | 51.866846603688714 |
| Pio | 90.12445887445887 |
| GW | 21.62162162162162 |
| GW+Pio | 40.0 |
### Chart: EB sizes
| Category | |
|---|---|
| DMSO | 227.4390158730159 |
| Pio | 278.18208485958485 |
| GW | 164.97875 |
| GW+Pio | 201.1592307692308 |* $
ns
*
$
#
#
#
*#$
D
E
### Chart: cTnT
| Category | |
|---|---|
| DMSO | 1.0 |
| Pio | 1.5800826237267582 |
| GW | 0.02393059370459977 |
| GW+Pio | 0.038 |
### Chart: a-MHC
| Category | |
|---|---|
| DMSO | 1.0 |
| Pio | 2.6944671537313867 |
| GW | 0.1672409443482638 |
| GW+Pio | 0.1975103279658438 |*
*
ns
ns
#
#
#
#
G
F
### Chart: SM22a
| Category | |
|---|---|
| DMSO | 1.7198699617421092 |
| Pio | 1.9393230339726817 |
| GW | 1.3390301572860037 |
| GW+Pio | 1.3214034352428126 |
### Chart: Pparg
| Category | |
|---|---|
| DMSO | 0.5 |
| Pio | 0.721309073195353 |
| GW | 0.41847932787365566 |
| GW+Pio | 0.42645146358872593 |*
ns
ns
#
#
#
#
